# Application of AP-MALDI Imaging Mass Microscope for the Rapid Mapping of Imipramine, Chloroquine, and Their Metabolites in the Kidney and Brain of Wild-Type Mice

**DOI:** 10.3390/ph15111314

**Published:** 2022-10-25

**Authors:** Ariful Islam, Takumi Sakamoto, Qing Zhai, Md. Muedur Rahman, Md. Al Mamun, Yutaka Takahashi, Tomoaki Kahyo, Mitsutoshi Setou

**Affiliations:** 1 Department of Cellular & Molecular Anatomy, Hamamatsu University School of Medicine, Hamamatsu, Shizuoka 431-3192, Japan; 2 Preppers Co., Ltd., Hamamatsu University School of Medicine, Hamamatsu, Shizuoka 431-3192, Japan; 3 International Mass Imaging Center, Hamamatsu University School of Medicine, Hamamatsu, Shizuoka 431-3192, Japan; 4 Department of Systems Molecular Anatomy, Institute for Medical Photonics Research, Preeminent Medical Photonics Education & Research Center, Hamamatsu, Shizuoka 431-3192, Japan

**Keywords:** MSI, visualization, DESI-MSI, MALDI-MSI, AP-MALDI-MSI, iMScope QT, imipramine, chloroquine, drug metabolites

## Abstract

Mass spectrometry imaging (MSI) is well-known for the non-labeling visualization of analytes, including drugs and their metabolites in biological samples. In this study, we applied three different tools of MSI, desorption electrospray ionization (DESI)-MSI, matrix-assisted laser desorption ionization (MALDI)-MSI, and a newly developed atmospheric pressure (AP)-MALDI-MSI known as iMScope^TM^ QT for rapid mapping of imipramine, chloroquine, and their metabolites in C57BL/6 male wild-type mice. Among three MSI tools, better detection capability for targeted drugs at higher speed (up to 32 pixels/s) was observed in iMScope QT. It revealed that imipramine and its metabolites were significantly accumulated in the renal cortex of mice, but chloroquine and its metabolites were highly accumulated in the renal pelvis and renal medulla of mice. Additionally, a higher accumulation of imipramine was noted in the thalamus, hypothalamus, septum, and hindbrain of mice brains. However, chloroquine and its metabolites showed notable accumulation in the lateral ventricle, fourth ventricle, and fornix of the mice brains. These findings of our study can be helpful in understanding clinically relevant properties, efficacy, and potential side effects of these drugs. Our study also showed the potentiality of iMScope QT for rapid mapping of small drugs and their metabolites in biological samples.

## 1. Introduction

Mass spectrometry imaging (MSI) techniques are continuously gaining popularity in various fields, including biological research, drug discovery, development, and delivery. It offers advantages for label-free imaging of analytes with semiquantitative and quantitative analysis [1]. It also simultaneously offers the detection of thousands of analytes and provides their spatial information at nm to µm spatial resolution present on the sample surface. The combination of detailed molecular composition and spatial distributions of detected analytes present on sample surfaces makes the MSI tools valuable for extensive characterization of the sample [1,2]. There are three common types of MSI tools: desorption electrospray ionization MSI (DESI-MSI), matrix-assisted laser desorption ionization MSI (MALDI-MSI), and secondary ion mass spectrometry (SIMS) imaging. Although SIMS imaging offers imaging of biomolecules at the nm scale in a lower *m*/*z* range, the high energy of the primary ion beam makes it challenging to detect intact molecular ions by SIMS. Therefore, DESI-MSI and MALDI-MSI are the most commonly used MSI tools in biological and pharmaceutical research for the imaging of intact molecular ions due to their soft-ionization and broad *m*/*z* range [3]. All of these tools have some advantages as well as disadvantages. Therefore, the selection of a suitable MSI tool is of utmost significance.

DESI-MSI is a recently developed MSI tool that offers the detection and localization of analytes present in the sample under ambient conditions [4]. It does not require sample pretreatment and unique glass slides such as MALDI-MSI. It shows better detection capability for small molecules such as free fatty acids, lipids, small peptides, and drug metabolites [5]. It uses a fine spray of charged droplets (solvents) on the sample surface to ionize molecules present on the sample surface and delivers them to a mass spectrometer as desolvated ions [6]. It is also non-destructive in nature, which makes it possible to acquire data from the same sample repeatedly when required. As it uses atmospheric pressure (AP) in the ionization source, it is also suitable for analyzing volatile molecules. On the other hand, MALDI-MSI is the most common tool of MSI, which also offers the detection, identification, and localization of proteins, lipids, drugs, metabolites, and small molecules present on the sample surface with higher sensitivity, specificity, and spatial resolution [7,8]. Additionally, the Fourier-transform ion cyclotron resonance (FT-ICR) mass analyzer equipped with the MALDI-MSI system offers very high mass accuracy [9]. Recently, there is an increasing interest in higher-resolution imaging with AP-MALDI-MSI for more focused beams. It makes sample preparation easier for analyzing volatile analytes in addition to other biomolecules at a higher spatial resolution [10,11]. It also offers an advantage for imaging analytes with a matrix that sublimes under a high vacuum used in traditional MALDI-MSI [12]. Imaging Mass Microscope (iMScope^TM^) is a tool of AP-MALDI-MSI equipped with a built-in optical microscope developed by Shimadzu, Japan. It is well known for speed and spatial resolution (up to 5 µm) [11]. Recently, they have developed another tool of AP-MALDI-MSI known as iMScope^TM^ QT for faster imaging with higher sensitivity and spatial resolution.

Imipramine is a well-known drug used to treat depression, anxiety, and nighttime bed-wetting. It inhibits serotonin and norepinephrine reuptake and blocks D2 receptors, antimuscarinic receptors, and adrenergic receptors [13]. Chloroquine is also a well-known drug used to treat viral diseases, mainly malaria and autoimmune diseases [14]. It inhibits the pH-dependent replication steps of several viruses. It reduces the production or release of tumor necrosis factor (TNF) α and interleukin 6 (IL-6), which are associated with inflammatory complications. It also shows preventive properties against COVID-19 [14]. Although these small drugs are widely used in human health care nowadays, both of these drugs have several side effects [15,16]. Localization information of these administered drugs and their metabolites in biological tissues is of paramount importance for better understanding their mode of action and toxic properties. However, there is still a lack of evidence about their localization in different organs. In this study, we tried rapid imaging of imipramine, chloroquine, and their metabolites for the first time in the mice kidney and brain using three different MSI tools: DESI-QTOF (DESI-MSI), 7T SolariX FT-ICR (MALDI-MSI) and iMScope QT (AP-MALDI-MSI). We applied three different MSI tools to select a suitable MSI tool for the rapid localization of those small drugs and their metabolites with higher sensitivity.

## 2. Results

### 2.1. Detection of Standard Drugs Applying Three Different MSI Tools Using Different Data Acquisition Speeds

**Figure 1:**
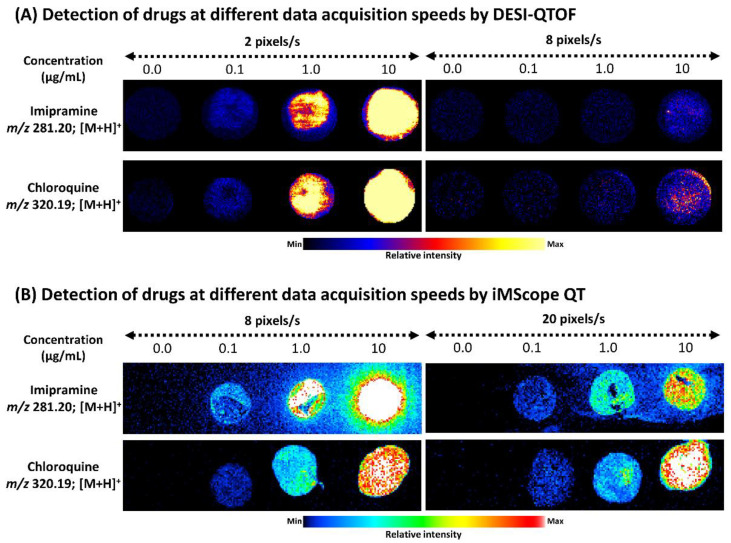
Ion images of imipramine and chloroquine detected by DESI-QTOF and iMScope QT. (**A**) Effects of data acquisition speed on the detection limit of DESI-QTOF for imipramine and chloroquine. (**B**) Effects of data acquisition speed on the detection limit of iMScope QT for imipramine and chloroquine. For DESI-QTOF, 0.3 µL solutions of both drugs were applied on a standard glass slide, and for iMScope QT, 0.3 µL solutions of both drugs were applied to the mice brain section. s: second.

After optimizing DESI source and data acquisition parameters, DESI-MSI data were acquired in positive ion mode over an *m*/*z* range of 150 to 550 from standard drugs applied on standard glass slides (0.3 µL/spot). We acquired DESI-MSI data at two different data acquisition speeds, 2 pixels/s and 8 pixels/s. DESI-MSI could detect both imipramine and chloroquine at 0.1 µg/mL concentration when data were acquired at 2 pixels/s. However, when we acquired DESI-MSI data at 8 pixels/s, we could detect both drugs at 10.0 µg/mL concentration (Figure 1A). In DESI-MSI, we observed that with increasing data acquisition speed, signal intensity for targeted drugs decreased drastically (Figure 1A and Figure S1A–D). 

We also optimized iMScope QT and acquired AP-MALDI-MSI data in positive ion mode from standard drugs applied to control mice brain section (0.3 µL/spot) at two different data acquisition speeds, 8 pixels/s and 20 pixels/s at the same *m*/*z* range. In iMScope QT, we detected both drugs at a 0.1 µg/mL concentration applying both data acquisition speeds (Figure 1B). Interestingly, data acquisition speed did not drastically affect the signal intensity of both drugs (Figure S1E–H).

MALDI-MSI data was also acquired in positive ion mode by 7T SolariX FT-ICR after optimizing its parameters from standard drugs applied to control mice brain section (0.3 µL/spot). Three different matrices and different methods were tried, but none of these drugs were detected in our experiments (Figure S1I,J).

### 2.2. Detection of Imipramine, Chloroquine, and Their Metabolites from the Treated Mice Kidneys

**Figure 2:**
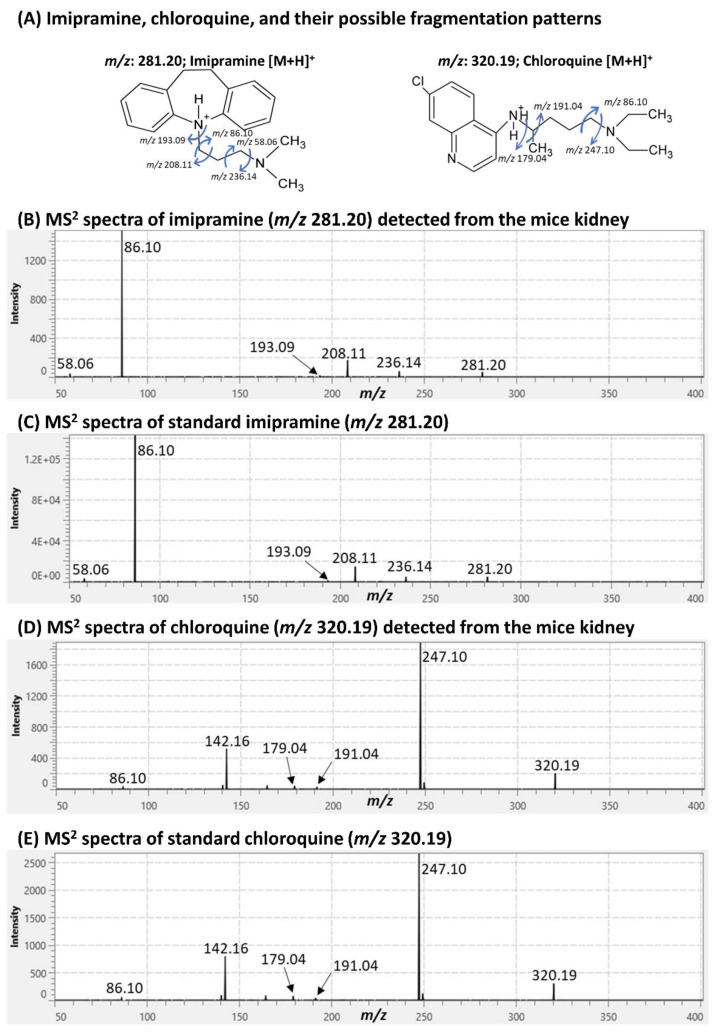
Tandem MS (MS^2^) spectra of imipramine and chloroquine acquired by iMScope QT from treated mice kidneys and standard drugs. (**A**) Possible fragmentation patterns of imipramine and chloroquine. (**B**,**C**) MS^2^ spectra of imipramine acquired from treated mice kidneys and standard imipramine. (**D**,**E**) MS^2^ spectra of chloroquine acquired from treated mice kidneys and standard chloroquine. α-Cyano-4-hydroxycinnamic acid (CHCA) was used as the matrix.

As we could not detect standard drugs by 7T SolariX FT-ICR, we acquired MSI data from control and treated mice kidney samples by applying DESI-QTOF (2 and 8 pixels/s) and iMScope QT (8 pixels/s). Similar to standards, both drugs were detected as protonated ions from treated mice samples by DESI-QTOF and iMScope QT (Figure S2). MS (*m*/*z*) peaks corresponded to imipramine, chloroquine, and their metabolites were not observed in the MSI data acquired from control mice kidneys (Figure S2). Imipramine was detected at *m*/*z* 281.20 [M+H]^+^, and chloroquine was detected at *m*/*z* 320.19 [M+H]^+^. For further confirmation of the detection of these drugs, we performed tandem mass spectrometry (MS^2^) imaging of selected *m*/*z* peaks corresponding to imipramine and chloroquine, applying iMScope QT (Figure 2).

MS^2^ spectra of *m*/*z* 281.20 showed similar product ions from standard imipramine and imipramine-treated mice kidneys (Figure 2A–C). These are the characteristic product ions of imipramine [17]. Similar to imipramine, MS^2^ spectra of *m*/*z* 320.19 showed identical product ions from standard chloroquine and chloroquine-treated mice kidneys (Figure 2A,D,E), hence assigned as chloroquine [18]. Product ion images also showed clear distribution in treated mice samples (Figure S3). Some metabolites of imipramine and chloroquine were also detected by DESI-QTOF and iMScope QT. Those metabolites were assigned based on their mass accuracy and previous reports (Table S1) [17,19]. Some of those metabolites were not detected by DESI-QTOF when acquiring data at the speed of 8 pixels/s (Table S2).

### 2.3. Localization of Imipramine, Chloroquine, and Their Metabolites in the Kidneys of Treated Mice Revealed by DESI-QTOF and iMScope QT Applying Different Data Acquisition Speed

**Figure 3:**
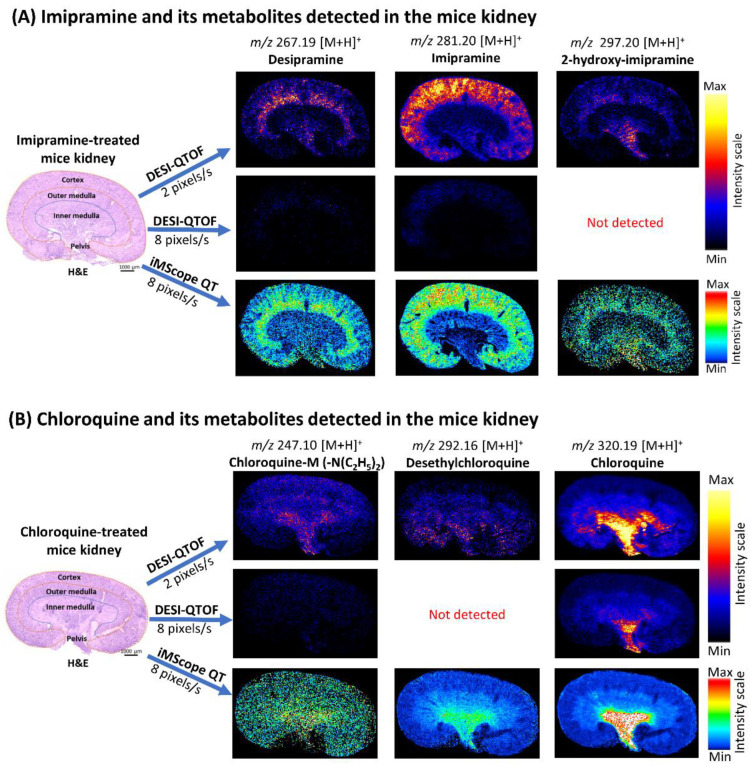
Localization of imipramine, chloroquine, and their metabolites in the kidney of treated mice applying DESI-QTOF and iMScope QT at different data acquisition speeds. (**A**) Distribution of imipramine and its metabolites in the treated mice kidney. (**B**) Distribution of chloroquine and its metabolites in the treated mice kidney. Here, spatial resolution was 50 µm × 50 µm (X,Y), and CHCA was used as the matrix for iMScope QT samples. s: second.

DESI-MSI data were acquired at the speeds of 2 pixels/s and 8 pixels/s by DESI-QTOF to confirm its capability to detect drugs and their metabolites at a higher speed (Figure 3A,B). AP-MALDI-MSI data were also acquired by iMScope QT at a speed of 8 pixels/s in our experiment (Figure 3A,B). This study revealed that DESI-QTOF could detect imipramine, chloroquine, and their several metabolites when acquired data at the speed of 2 pixels/s, but at a higher speed (8 pixels/s), it detected both drugs and some of their metabolites with lower signal intensity. Drug metabolites with lower abundance were not detected by DESI-QTOF at higher speed (Figure 3A,B, and Table S2). On the other hand, iMScope QT detected both drugs and more metabolites at a higher speed (8 pixels/s) with higher signal intensity (Figure 3A,B, and Table S2). Additionally, to acquire data at the same data acquisition speed (8 pixels/s), DESI-MSI required more time than iMScope QT due to having movement of the DESI 2D stage after scanning each row. For the mice kidney section, a measuring area with 32802 data points (pixel size 50 µm × 50 µm, X,Y), DESI-QTOF required 86 minutes at 8 pixels/s, whereas iMScope QT required 68 minutes at the same data acquisition speed. Both MSI tools revealed the localization of imipramine, chloroquine, and their metabolites in the mice kidneys for the first time. Imipramine and its metabolites were mainly accumulated in the cortex (renal cortex), outer medulla (renal medulla), and a small amount in the pelvis (renal pelvis). Chloroquine and its metabolites were mainly accumulated in the pelvis and inner medulla of the mice kidneys. None of these drugs and their metabolites were detected in the kidney of control mice by DESI-QTOF and iMScope QT (Figure S4).

### 2.4. Localization of Imipramine, Chloroquine, and Their Metabolites in the Brain of Treated Mice Revealed by iMScope QT

**Figure 4:**
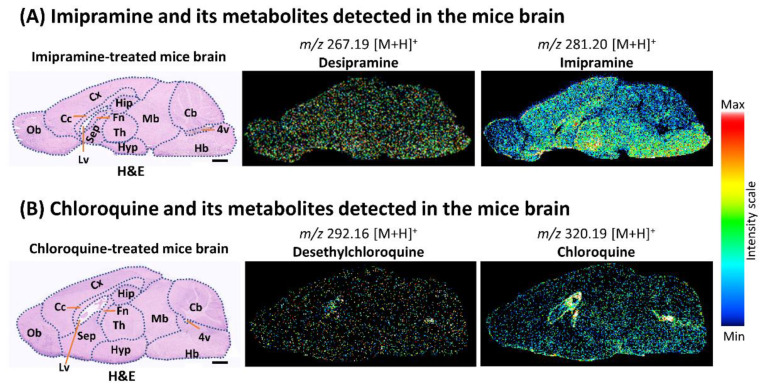
Localization of imipramine, chloroquine, and their metabolites in the brain of treated mice applying iMScope QT. (**A**) Distribution of imipramine and its metabolites in the mice brain. (**B**) Distribution of chloroquine and its metabolites in the mice brain. Cb: cerebellum; Hb: hindbrain; Mb: midbrain; Hip: hippocampus; Cx: cerebral cortex; Th: thalamus; Fn: fornix; Cc: corpus callosum; Ob: olfactory bulb; Sep: septum; Hyp: hypothalamus; Lv: lateral ventricle; 4v: fourth ventricle; Scale bar: 1000 µm.

As DESI-QTOF could not detect some metabolites of imipramine and chloroquine in the kidney at a higher data acquisition speed, we acquired MSI data from mice brains samples only by iMScope QT (8 pixels/s). Similar to kidney samples, iMScope QT revealed the localization of both drugs and their metabolites in treated mice brains for the first time (Figure 4 and Figure S5). Imipramine and its metabolites were accumulated throughout the brain of the treated mice, but a higher accumulation of imipramine was observed in the thalamus, hypothalamus, septum, and hindbrain regions (Figure 4A). Interestingly, chloroquine and its metabolites were highly accumulated in the lateral ventricle, fourth ventricle, and fornix of the treated mice brain (Figure 4B). 

### 2.5. Rapid Mapping of Imipramine, Chloroquine, and Their Metabolites with Higher Spatial Resolution Applying iMScope QT

**Figure 5:**
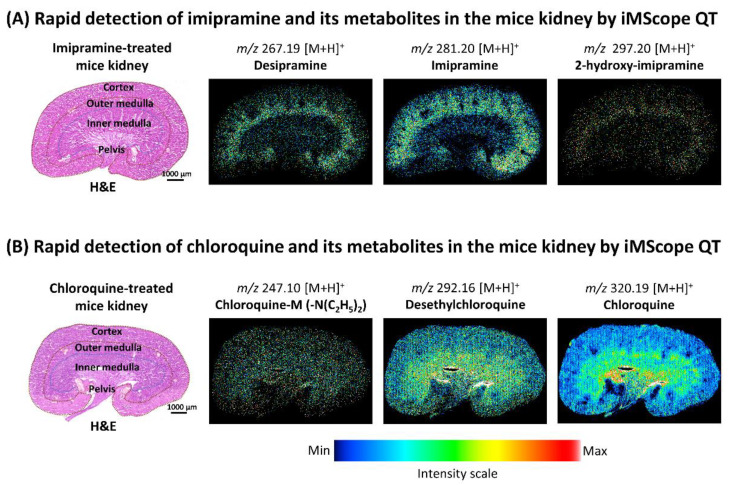
Rapid localization of imipramine, chloroquine, and their metabolites in the kidneys of treated mice with higher spatial resolution applying iMScope QT. (**A**) Distribution of imipramine and its metabolites in the mice kidney using higher data acquisition speed (32 pixels/s). (**B**) Distribution of chloroquine and its metabolites in the mice kidney using higher data acquisition speed (32 pixels/s). Here, the spatial resolution was 25 µm × 25 µm (X,Y).

As higher data acquisition speed (8 and 20 pixels/s) in iMScope QT did not drastically affect the signal intensity, we tried a higher data acquisition speed (32 pixels/s) for rapid localization of imipramine, chloroquine, and their metabolites in treated mice kidneys with a higher spatial resolution (pixel size 25 µm × 25 µm, X,Y). Even acquiring data rapidly, iMScope QT detected both drugs and all metabolites, which were detected at 8 pixels/s (Figure 5A,B). Additionally, we observed a similar distribution of drugs and their metabolites in the treated mice kidneys, even applying rapid data acquisition speed.

## 3. Discussion

In this study, we revealed the localization of imipramine, chloroquine, and their metabolites in the mice kidneys and brains by applying different tools of MSI. We tried DESI-QTOF, iMScope QT, and 7T SolariX FT-ICR to select the suitable one for rapidly imaging these drugs and their metabolites. We observed better signal intensity and sensitivity of iMScope QT at a higher data acquisition speed for our targeted drugs and their metabolites. Thereafter, we revealed the localization of administrated imipramine, chloroquine, and their metabolites using iMScope QT in the kidneys and brains of treated mice. To the best of our knowledge, this is the first study about the localization of imipramine, chloroquine, and their metabolites in mice.

DESI-MSI (DESI-QTOF) is widely used nowadays for the detection and localization of small biomolecules, drugs, and metabolites under ambient conditions with minimum sample preparation [20]. Due to not using matrix molecules such as MALDI-MSI, it is also free from ion suppression due to matrix-derived peaks at lower *m*/*z* regions [21]. It can detect analytes as intact molecular ions due to not using ion beams or laser power [22]. After optimizing DESI-MSI parameters, we acquired DESI-MSI data at the usual speed (2 pixels/s) [5] and a little higher speed (8 pixels/s) from standard imipramine and chloroquine. At the typical data acquisition speed, DESI-MSI detected both drugs with very good sensitivity up to 0.1 µg/mL concentration. However, the signal intensity of DESI-MSI for both drugs drastically reduced when we acquired data at a higher data acquisition speed (8 pixels/s). At higher data acquisition speed, lower detectable concentration by DESI-MSI was 10 µg/mL for both drugs. AP-MALDI-MSI is also well known for having several advantages for detecting volatile and small molecules from sample surfaces [10]. In our current study, we applied a newly developed tool of AP-MALDI-MSI Imaging Mass Microscope known as iMScope QT for rapid detection of imipramine and chloroquine with higher sensitivity. This tool is developed by Shimadzu, Japan, for rapid measurement with higher spatial resolution. In our study, iMScope QT detected both drugs at higher speeds (8 pixels/s and 20 pixels/s) with sensitivity up to 0.1 µg/mL concentration. Even 20 pixels/s data acquisition speed did not affect signal intensity so much. We also used 7T SolariX FT-ICR (MALDI-MSI) to detect targeted drugs at a higher speed. Using the same matrix as iMScope QT (0.7 µm thick CHCA) at the usual data acquisition speed (1.36 pixels/s), it could not detect either imipramine or chloroquine at 10 µg/mL concentration, which was the maximum concentration used in DESI-MSI and AP-MALDI-MSI with iMScope QT. We tried three matrices, CHCA, 2,5-Dihydroxybenzoic acid (DHB), and 9-Aminoacridine (9AA), with different methods using iMLayer and TM-Sprayer to apply matrix for MALDI-MSI [23,24,25], but could not detect targeted drugs. Maybe higher concentrations of those drugs or other sample pretreatment methods were required for their detection by MALDI-MSI.

We could not detect imipramine and chloroquine by MALDI-MSI, so we acquired DESI-MSI and AP-MALDI-MSI data from treated and control mice kidneys using 50 µm pixel size (spatial resolution). Both MSI tools detected targeted drugs and their metabolites in treated mice kidneys. DESI-MSI data acquired at 2 and 8 pixels/s revealed the localization of both drugs and some of their metabolites in the mice kidneys. We also noted that the signal intensity of DESI-MSI for both drugs and their metabolites notably reduced at higher data acquisition speed (8 pixels/s), and even drug metabolites with lower abundance were not detected. On the other hand, acquiring data at 8 pixels/s speed, iMScope QT detected both drugs and several metabolites with higher signal intensity and precise distribution. Applying DESI-MSI and iMScope QT, we revealed that imipramine was highly accumulated in the renal cortex, and its metabolites desipramine, 2-hydroxy-desipramine, and 2-hydroxy-imipramine glucuronide were accumulated in the outer renal medulla and renal pelvis of treated mice. We also found that chloroquine, desethylchloroquine, and chloroquine-M (-N(C_2_H_5_)_2_) were accumulated mainly in the pelvis and in small amounts in the inner medulla of treated mice kidneys. The renal cortex is the outer layer of the kidney where the nephrons begin. It connects blood vessels to the nephrons. It is also associated with the production of red blood cells. The lower part of the kidney is known as the renal medulla. It can be divided into the outer and inner medulla. It consists of renal pyramids, which contain a dense network of nephrons, functional units of the kidney. The renal pelvis is positioned in the central region of the kidney. It is continuous with the ureter, which collects urine from the renal pyramid. All these parts of the kidneys play essential roles in filtering blood, maintaining electrolyte balance, and removing waste products from the body. As kidneys are commonly exposed to drugs and their metabolites present in blood, drug-induced toxicity in the kidney is common worldwide [26,27]. Accumulated imipramine and its metabolites in the kidney observed in our study can show toxic effects by inducing glomerulonephritis and inflammatory cell infiltration [28]. They also can reduce antioxidant levels and cause oxidative damage of the kidney [28]. Additionally, chloroquine and its metabolites observed in our study also might be associated with renal damage. They can cause oxidative damage by increasing lipid peroxidation and decreasing antioxidant enzyme activities [29,30]. However, further research is required to explore the detailed toxic effects of those accumulated drugs and their metabolites in specific kidney regions observed in our study.

Due to the detection of a few drug metabolites by DESI-MSI at a higher data acquisition speed (8 pixels/s), we applied only iMScope QT to reveal the localization of both drugs and their metabolites in the brain of mice. We observed the localization of imipramine and its metabolites throughout the brain, but a little higher amount of imipramine was observed in the thalamus, hypothalamus, septum, and hindbrain of treated mice. Imipramine is commonly used to treat anxiety and depression [31]. According to previous reports, accumulated imipramine in these brain regions might inhibit the reuptake of serotonin and norepinephrine and help to relieve anxiety and depression [31,32]. Our study also explored a considerable accumulation of chloroquine and its metabolites in the lateral ventricle, fourth ventricle, and fornix of treated mice brains. The choroid plexus in the lateral ventricle and fourth ventricle produces cerebrospinal fluid, which plays a vital role in maintaining central nervous system homeostasis [33,34]. It regulates the volume of the brain and cranial cavity, eliminates waste metabolites and unnecessary substances, and transports proteins, nutrients, and drugs [34,35]. The fornix, a bundle of white matter, plays a critical role in cognitive function by connecting several nodes of a limbic circuitry [36]. Imipramine and its metabolites increase the production of inflammatory cytokines (IL 1-β and TNF- α) and nitric oxide from microglia [16]. Chloroquine and its metabolites are also known for neurotoxic effects such as psychosis, seizure, disorientation, and hallucination [15,37]. As both imipramine and chloroquine can cause neurotoxicity, their accumulation in different brain regions observed in this study might be responsible for the neurotoxic effects and disruption of their functions. Further studies are required to explore the details mechanism of action of these accumulated drugs and drug metabolites associated with brain function and health.

In this study, we also observed that acquiring data at a higher speed (20 pixels/s) by newly developed iMScope QT does not affect the sensitivity much. Therefore, we tried to visualize imipramine, chloroquine, and their metabolites at a higher data acquisition speed (32 pixels/s) and higher spatial resolution (25 µm × 25 µm; X,Y) using iMScope QT. At this speed and spatial resolution, we detected both drugs and most of their metabolites from treated mice kidneys by applying iMScope QT with a clear distribution. Therefore, this MSI tool can be a good option for users to perform rapid imaging with a higher spatial resolution of drugs, metabolites, and other analytes. However, this time we used only two drugs, imipramine and chloroquine. For other compounds, including the drugs, MALDI-MSI and DESI-MSI may offer much better sensitivity compared to iMScope QT. In the future, we will try other compounds and drugs for rapid imaging with a higher spatial resolution by applying different MSI tools.

## 4. Materials and Methods

### 4.1. Animals

We purchased four-month-old C57BL/6JJmsSlc wild-type male mice (weight 26–28 gm) from SLC Inc. (Hamamatsu, Japan). We reared all mice at 12 hours of light/dark cycle for one week with ad libitum access to food and water. After one week, all mice were randomly divided into three groups (*n* = 3 for each group). Mice of group I were injected (intraperitoneal; IP) with normal saline water. Mice of groups II and III received IP injections of imipramine and chloroquine (30 mg/kg, dissolved in normal saline water), respectively. After two hours of IP injection, all mice were dissected following cervical dislocation, and kidney and brain samples were collected rapidly. Thereafter, all samples were quickly frozen into dry ice. Then, frozen samples were stored at −80 °C until preparing sections.

### 4.2. Chemicals and Reagents

LC/MS grade methanol, ethanol, 2-propanol, ultrapure water, acetonitrile, and formic acid were purchased from Wako Pure Chemical Industries (Osaka, Japan). Trifluoroacetic acid (TFA), sodium formate, leucine enkephalin, 9AA, and CHCA were purchased from Sigma A Sigma-Aldrich (St. Louis, MO, USA). DHB was purchased from Bruker Daltonics (Bremen, Germany).

### 4.3. Preparation of Tissue Section for MSI Measurements

Sagittal sections of mice kidneys and brains were prepared according to the previously described method [38] using CM1950 cryostat (Leica Biosystems, Wetzlar, Germany). For DESI-MSI, 10 μm thick sections were mounted on uncoated glass slides (Matsunami, Osaka, Japan), and 10 μm thick sections were mounted on Indium Tin Oxide (ITO) coated glass slides (100 Ω, Matsunami, Osaka, Japan) for MALDI-MSI and AP-MALDI-MSI.

### 4.4. Standard Solution Preparation

For MSI measurements, standard imipramine and chloroquine were dissolved in 80% ethanol and 80% acetonitrile, respectively, and prepared stock solutions with 0.0, 0.1, 1.0, and 10.0 μg/mL concentrations.

### 4.5. DESI-MSI Analysis

DESI-MSI data were acquired applying Xevo G2-XS quadrupole time of flight (Q-TOF) mass spectrometer (Waters, Milford, MA, USA) coupled to a 2D-DESI source. After calibrating mass spectra and optimizing the signal intensity of DESI-QTOF following the previous method [39], DESI-MSI data was acquired from standard drugs applied on glass slides (0.3 μL/spot) to confirm the detection. Thereafter, DESI-MSI data were acquired from mice kidney and brain sections in positive ion mode at an *m*/*z* range of 150 to 550. All parameters used for the optimization of DESI-MSI and data acquisition are given in Table S3.

### 4.6. AP-MALDI-MSI Analysis Using iMScope QT

Sagittal sections of mice kidneys and brains mounted on ITO-coated glass slides were dried before applying the matrix. Then, CHCA was heated at 250 °C and deposited (0.7 µm) on the sample surface by applying iMLayer^TM^ (Shimadzu, Japan). The iMLayer chamber was maintained at vacuum conditions during the matrix deposition. Thereafter, MSI data was acquired by iMScope QT (Imaging Mass Microscope, Shimadzu, Japan) from mice samples and standard drugs applied (0.3 μL/spot) on control mice brain sections. In iMScope QT, an atmospheric pressure matrix-assisted laser desorption/ionization (AP-MALDI) source was equipped with LCMS-9030 Q-TOF mass spectrometer. Prior to measuring mice samples, iMScope QT parameters were optimized using standard drugs. Parameters used to acquire data by iMScope QT were given in Table S4. For the confirmation of the detection of targeted drugs from mice samples, we performed MS^2^ imaging of standard drugs and treated mice samples by iMScope QT. For MS^2^ imaging of both drugs, 35 V as collision energy was used, and all other parameters were same as described for MSI data acquired at 8 pixels/s (Table S4). 

### 4.7. MALDI-MSI Analysis Using 7T SolariX FT-ICR

Prior to acquiring MALDI-MSI data (vacuum), treated mice brain and kidney sections and standard drugs applied control mice brain sections (0.3 µL/spot) were dried in a desiccator at room temperature. Then, three different matrices were deposited on the sample surface using iMLayer (Shimadzu, Japan) and HTX TM-Sprayer^TM^ (HTX Technologies, Chapel Hill, NC, USA). CHCA, DHB, and 9AA were deposited on the sample surface by iMLayer at a thickness of 0.7 µm, 1.5 µm, and 1.0 µm, respectively. CHCA, DHB, and 9AA were also sprayed on the sample surface by HTX TM-Sprayer according to previously reported methods [23,24,25]. After matrix application, we acquired MALDI-MSI data in positive ion mode by applying 7T SolariX FT-ICR equipped with a superconducting magnet (7.0 T) and a Smartbeam II™ laser (355 nm) unit (Bruker Daltonics). Laser focus was set to small, and laser pulses were set to 1000 Hz. Other major parameters used for the data acquisition were as follows; laser shots 100, laser power 30 to 50%, *m*/*z* range 150 to 550, mass resolution 1M, and time of flight 0.6 to 0.8 millisecond. Different laser power and time of flight were tried for method optimization.

### 4.8. MSI Data Analysis

DESI-MSI data was acquired and processed by MassLynx 4.1 (Waters, Milford, MA, USA) software. HDImaging 1.4 (Waters, Milford, MA, USA; version 1.4) software was used for constructing 2D ion images and distribution analysis of candidate ions. Imaging MS Solution (Shimadzu, Japan, version 2.00.00A) software was used to acquire AP-MALDI-MSI data by iMScope QT, and IMAGE REVEAL^TM^ MS (Shimadzu, Japan, version 1.20.0.10960) software was used to analyze the iMScope QT data. Vacuum MALDI-MSI (SolariX FT-ICR) data were acquired using ftmsControl (Bruker Daltonics, version 2.3.0) and analyzed by fleximaging (Bruker Daltonics, version 5.0). All experiments were performed at least three times to confirm the biological (with different mice of the same groups) and instrumental reproducibility (standard drugs).

## 5. Conclusions

For the first time, we revealed the localization of imipramine, chloroquine, and their metabolites in the mice kidney and brain by applying a newly developed AP-MALDI-MSI known as iMScope QT, which offered rapid data acquisition with higher sensitivity. The findings of our study might be helpful in exploring the mode of action and toxic properties of these drugs. Additionally, our study also represents the potentiality of iMScope QT for rapid imaging of analytes present in the sample.

## Data Availability

Data are contained within the article and Supplementary Material.

## Supplementary Materials

The following supporting information can be downloaded at: https://www.mdpi.com/article/10.3390/ph15111314/s1, Figure S1: MS spectra of imipramine and chloroquine acquired by DESI-QTOF, iMScope QT, and 7T SolariX FT-ICR at different speeds; Figure S2: Representative MS spectra acquired by DESI-QTOF and iMScope QT from sagittal sections of mice kidneys; Figure S3: Tandem MS (MS^2^) imaging of imipramine and chloroquine in treated mice kidneys by iMScope QT; Table S1: Metabolites of imipramine and chloroquine detected from treated-mice kidneys by DESI-QTOF and iMScope QT; Table S2: List of the metabolites of the imipramine and chloroquine detected from mice kidneys by DESI-QTOF and iMScope QT applying different data acquisition speeds; Figure S4: Ion images of imipramine, chloroquine, and their metabolites in the kidney of control mice applying DESI-QTOF and iMScope QT; Figure S5: Ion images of imipramine, chloroquine, and their metabolites in the brain of control mice applying iMScope QT; Table S3: Parameters used for the optimization and data acquisition by DESI-QTOF, Table S4: Parameters used for the optimization and data acquisition by iMScope QT.
