# Supplementary material for: Application of AP-MALDI Imaging Mass Microscope for the Rapid Mapping of Imipramine, Chloroquine, and Their Metabolites in the Kidney and Brain of Wild-Type Mice
Source: Pharmaceuticals (Basel). 2022 Oct 25;15(11):1314. doi: 10.3390/ph15111314 (PMC9696843; doi:10.3390/ph15111314)
Supplement: Supplementary file 1 [file pharmaceuticals-15-01314-s001.zip › pharmaceuticals-1963640-Supplementary materials.pdf]

Supplementary materials

Communication

Application of AP-MALDI Imaging Mass Microscope for the Rapid Mapping of Imipramine, Chloroquine, and Their Metabolites in the Kidney and Brain of Wild-Type Mice

Ariful Islam[1,2,*], Takumi Sakamoto[1,2], Qing Zhai[1], Md. Muedur Rahman[1], Md. Al Mamun[1,2], Yutaka Takahashi[1,2,3], Tomoaki Kahyo[1,2], Mitsutoshi Setou[1,3,4]

[1] Department of Cellular & Molecular Anatomy, Hamamatsu University School of Medicine, Hamamatsu 431-3192, Shizuoka, Japan
[2] Preppers Co. Ltd., Hamamatsu 431-3192, Shizuoka, Japan
[3] International Mass Imaging Center, Hamamatsu University School of Medicine, Hamamatsu 431-3192, Shizuoka, Japan
[4] Department of Systems Molecular Anatomy, Institute for Medical Photonics Research, Preeminent Medical Photonics Education & Research Center, Hamamatsu 431-3192, Shizuoka, Japan
* Correspondence: arif5219@hama-med.ac.jp; Tel.: +81-53-435-2086; FAX: +81-53-435-2468.

Figure S1

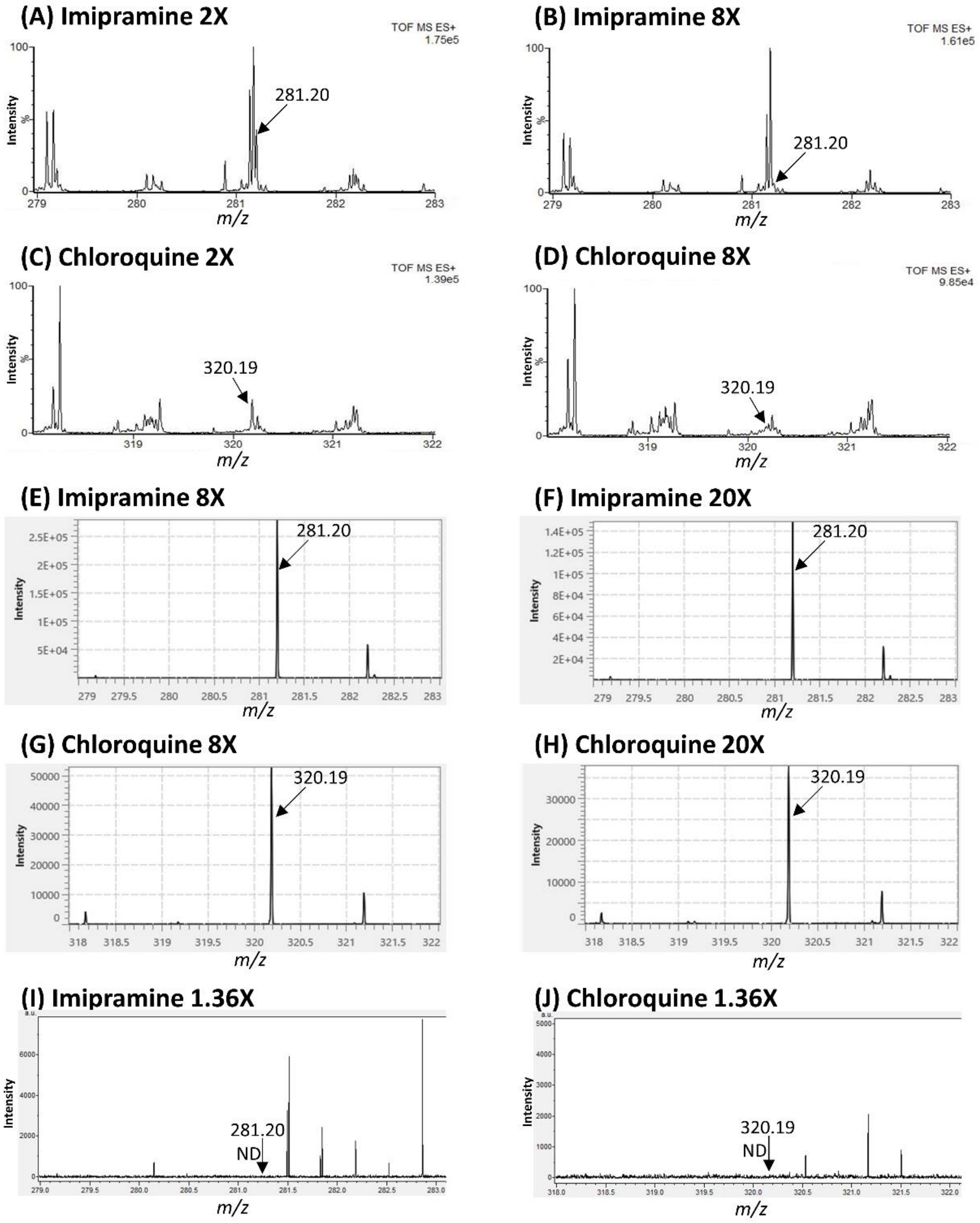

Figure S1: MS spectra of imipramine and chloroquine acquired by DESI-QTOF, iMScope QT, and 7T SolariX FT-ICR at different speeds. (A-D) Represent MS spectra of standard imipramine and chloroquine acquired by DESI-QTOF at different data acquisition speeds. (E-H) Represent MS spectra of standard imipramine and chloroquine obtained by iMScope QT at different data acquisition speeds. (I-J) Represent MS spectra of standard imipramine and chloroquine acquired by 7T SolariX FT-ICR. For DESI-QTOF, 0.3 μL solutions of both drugs (10 μg/mL) were applied on a standard glass slide. For iMScope QT and SolariX FT-ICR, 0.3 μL solutions of both drugs (10 μg/mL) were applied to mice brain sections. Then 0.7 μm thick CHCA matrix was deposited by iMLayer and used for MALDI-MSI (7T SolariX FT-ICR) and AP-MALDI-MSI (iMScope QT) data acquisition. Here, 1.36X. 2X, 8X, and 20X indicate the number of pixels scanned per second (s). ND: not detected.

Figure S2

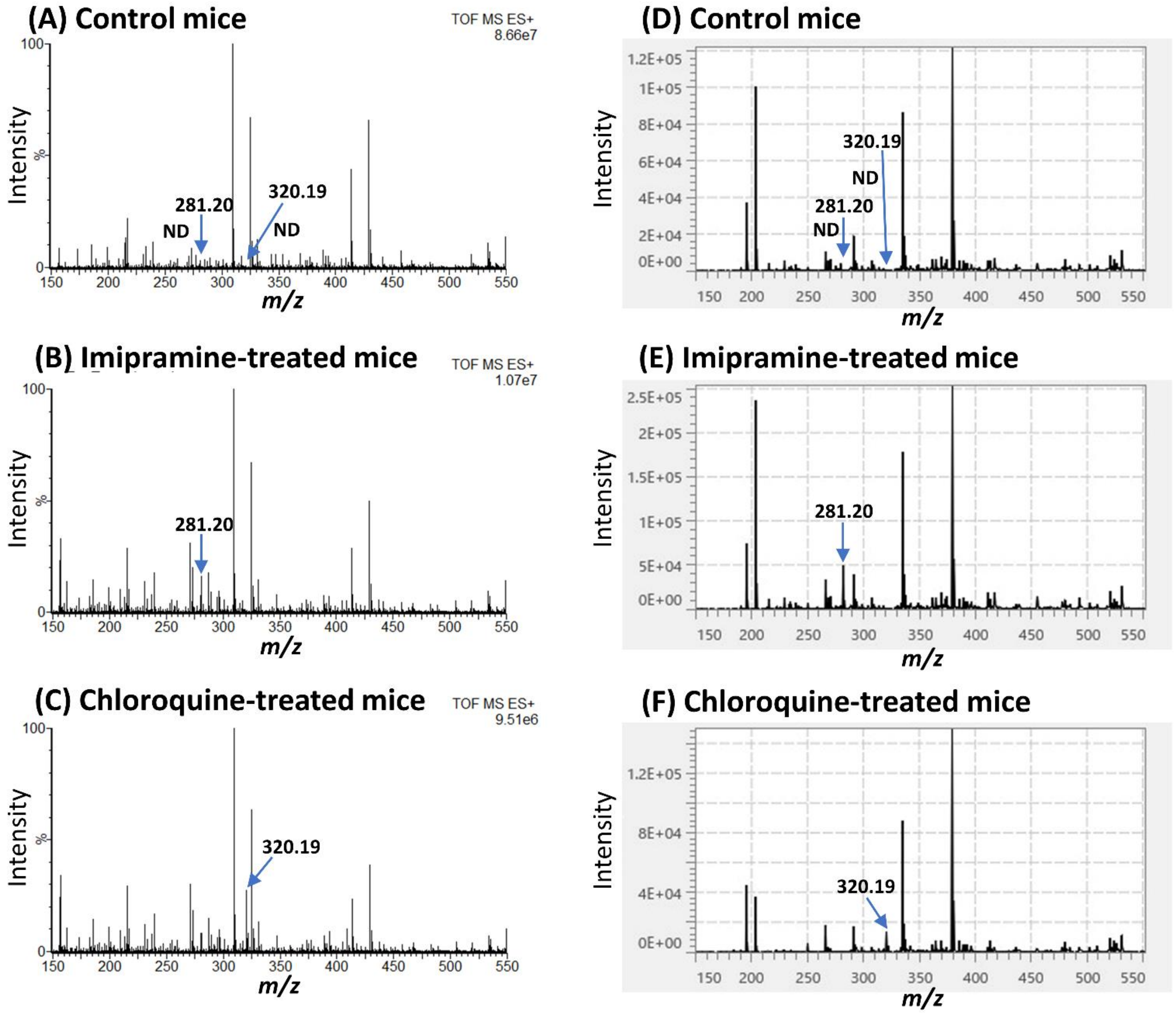

Figure S2: Representative MS spectra acquired by DESI-QTOF and iMScope QT from sagittal sections of mice kidneys. (A-C) Represent DESI-QTOF MS spectra obtained from control, imipramine, and chloroquine-treated mice kidney sections, respectively. (D-F) Represent iMScope QT MS spectra acquired from control, imipramine, and chloroquine-treated mice kidney sections, respectively. *m/z* 281.20: protonated ion of imipramine, and *m/z* 320.19: protonated ion of chloroquine. Data acquisition speed for DESI-QTOF was 2 pixels/s, and for iMScope QT was 8 pixels/s.

Figure S3

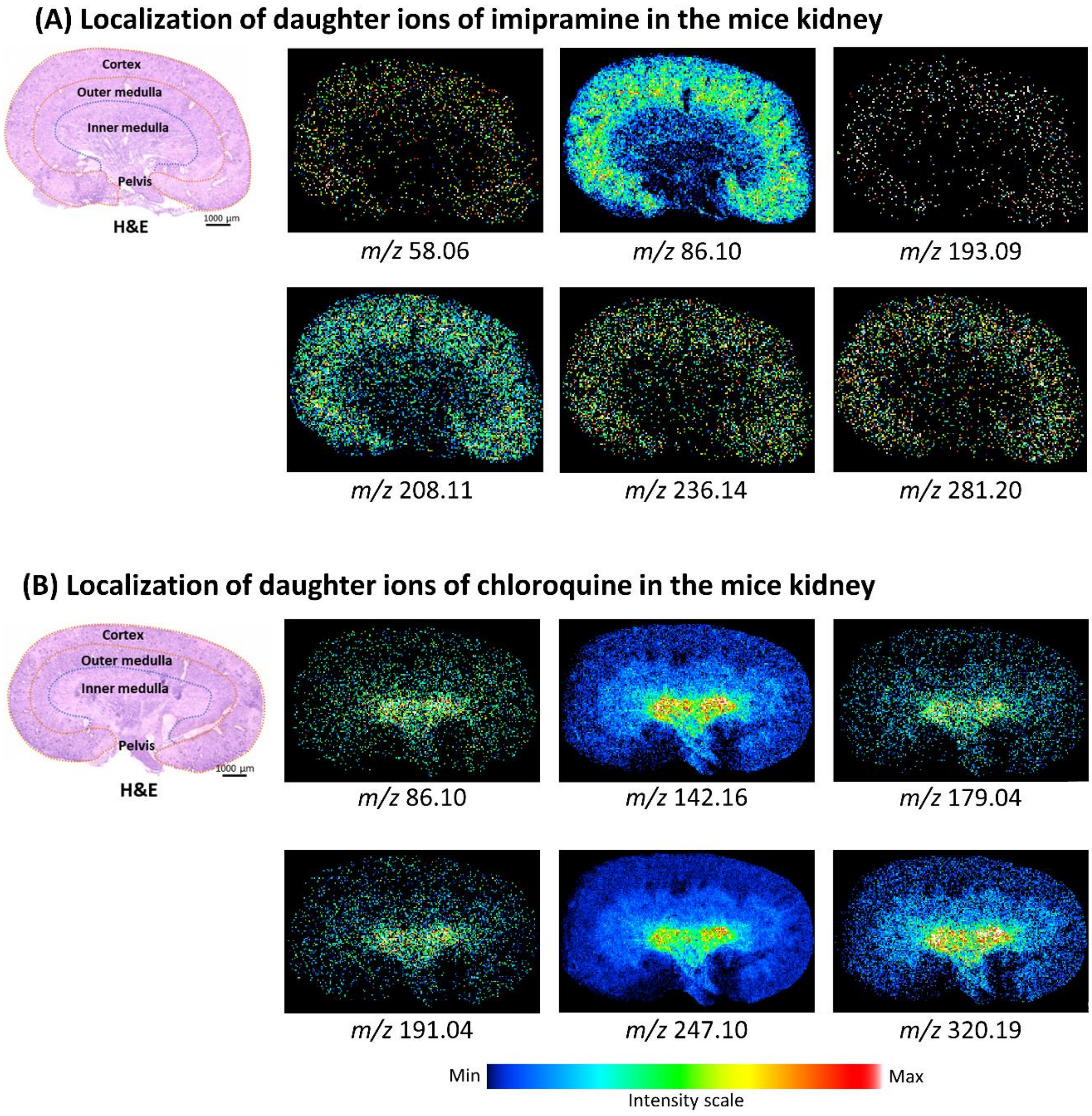

Figure S3: Tandem MS (MS^2) imaging of imipramine and chloroquine in treated mice kidneys by iMScope QT. (A) Product ion images of imipramine in treated-mice kidneys. (B) Product ion images of chloroquine in treated-mice kidneys. Here, spatial resolution was 50 µm x 50 µm (X,Y), and CHCA was used as the matrix for iMScope QT samples.

Table S1: Metabolites of imipramine and chloroquine detected from treated-mice kidneys by DESI-QTOF and iMScope QT.

Mice groups	Candidate molecules	Theoretical mass	DESI-QTOF (2 pixels/s)		iMScope QT (8 pixels/s)	
			Observed mass	Mass error (ppm)	Observed mass	Mass error (ppm)
**Imipramine treated-mice**	Desdimethyl-imipramine	275.1519 $[M+Na]^+$	ND	-	275.1513	2
	Desipramine	267.1856 $[M+H]^+$	267.1910	20	267.1875	7
	2-hydroxy-imipramine	297.1961 $[M+H]^+$	297.1990	10	297.1974	4
	2-hydroxy-imipramine glucuronide	473.2282 $[M+H]^+$	ND	-	473.2322	8
**Chloroquine treated-mice**	Chloroquine-M (-$N(C_2H_5)_2$)	247.1002 $[M+H]^+$	247.0965	15	247.1030	11
	Desethylchloroquine	292.1575 $[M+H]^+$	292.1572	1	292.1553	8

ND: not detected; s: second.

Table S2: List of the metabolites of the imipramine and chloroquine detected from mice kidneys by DESI-QTOF and iMScope QT applying different data acquisition speeds.

**Imipramine and its metabolites**			
**Candidates**	**DESI-QTOF (2 pixels/s)**	**DESI-QTOF (8 pixels/s)**	**iMScope QT (8 pixels/s)**
Desdimethyl-imipramine	Not detected	Not detected	Detected $[M+Na]^+$
Desipramine	Detected $[M+H]^+$	Detected $[M+H]^+$	Detected $[M+H]^+$
Imipramine	Detected $[M+H]^+$	Detected $[M+H]^+$	Detected $[M+H]^+$
2-hydroxy-imipramine	Detected $[M+H]^+$	Not detected	Detected $[M+H]^+$
2-hydroxy-imipramine glucuronide	Not detected	Not detected	Detected $[M+H]^+$
**Chloroquine and its metabolites**			
**Candidates**	**DESI-QTOF (2 pixels/s)**	**DESI-QTOF (8 pixels/s)**	**iMScope QT (8 pixels/s)**
Chloroquine-M ($-N(C_2H_5)_2$)	Detected $[M+H]^+$	Detected $[M+H]^+$	Detected $[M+H]^+$
Desethylchloroquine	Detected $[M+H]^+$	Not detected	Detected $[M+H]^+$
Chloroquine	Detected $[M+H]^+$	Detected $[M+H]^+$	Detected $[M+H]^+$

s: second.

Figure S4

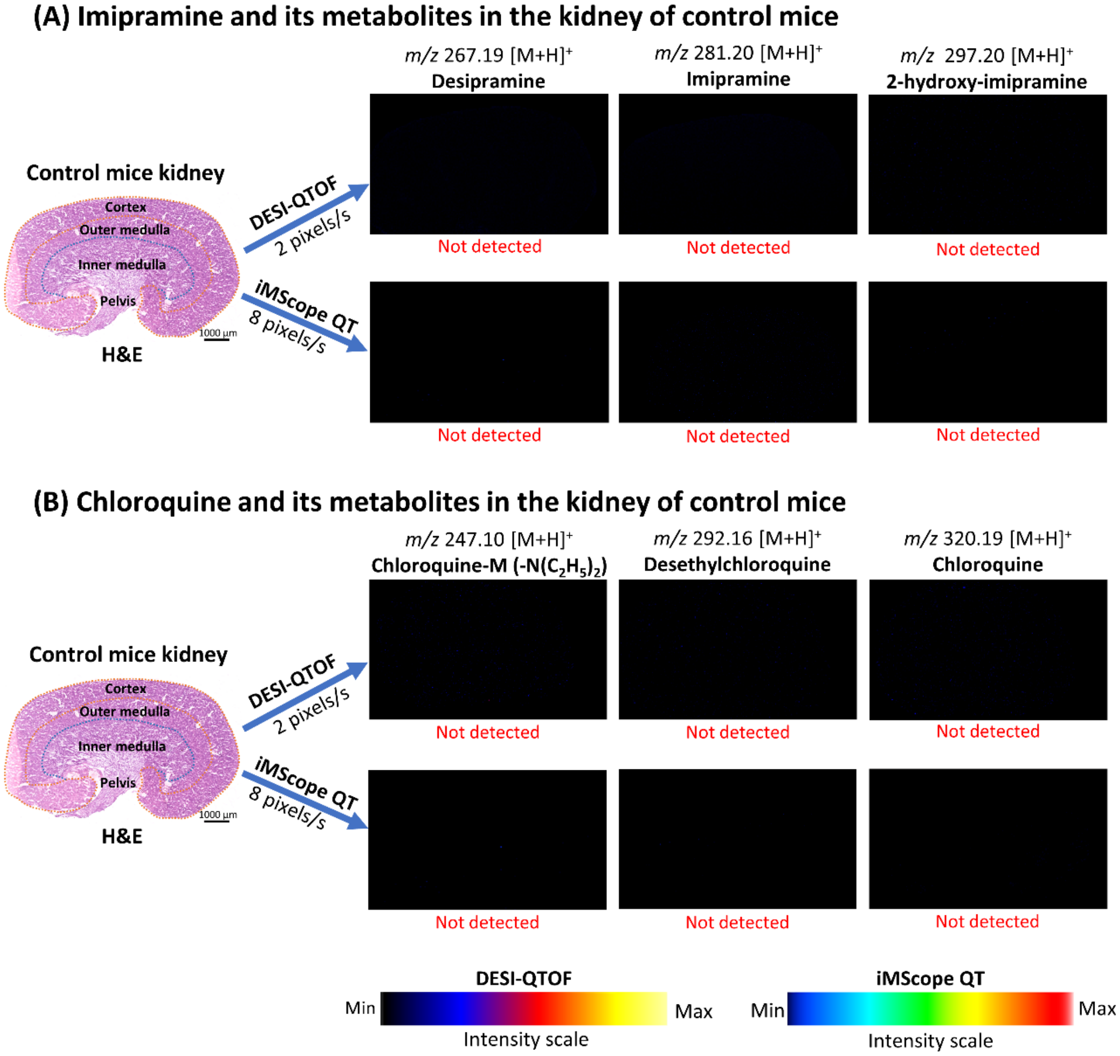

Figure S4: Ion images of imipramine, chloroquine, and their metabolites in the kidney of control mice applying DESI-QTOF and iMScope QT. (A) Ion images of imipramine and its metabolites in the kidney of control mice. (B) Ion images of chloroquine and its metabolites in the kidney of control mice. Here, spatial resolution was 50 µm x 50 µm (X,Y), and CHCA was used as the matrix for iMScope QT samples. None of these drugs and their metabolites were detected in the control mice kidney. s: second.

Figure S5

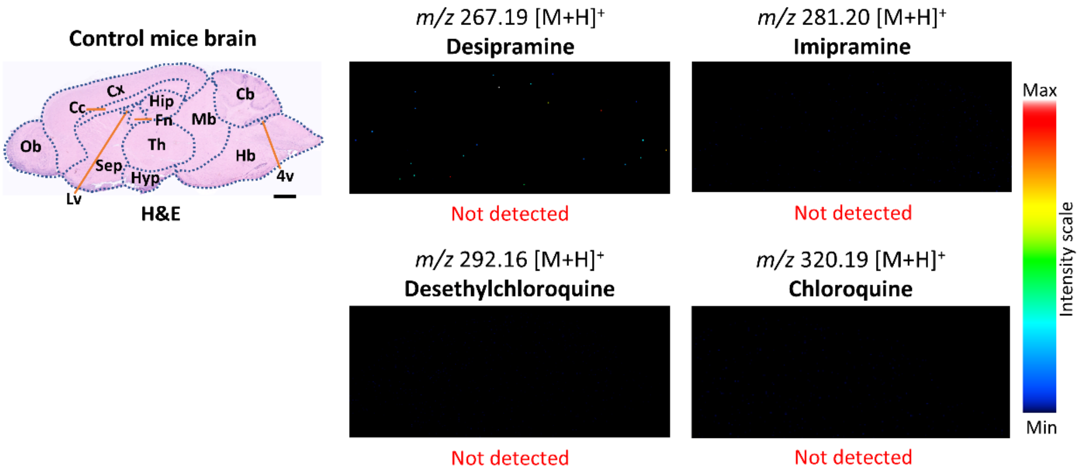

Figure S5: Ion images of imipramine, chloroquine, and their metabolites in the brain of control mice applying iMScope QT. Cb: cerebellum; Hb: hindbrain; Mb: midbrain; Hip: hippocampus; Cx: cerebral cortex; Th: thalamus; Fn: fornix; Cc: corpus callosum; Ob: olfactory bulb; Sep: septum; Hyp: hypothalamus; Lv: lateral ventricle; 4v: fourth ventricle; Scale bar: 1000 µm. Imipramine, chloroquine, and their metabolites were not detected in the brain of control mice.

Table S3: Parameters used for the optimization and data acquisition by DESI-QTOF.

**Ion source parameters**	Capillary voltage	3.0 kV
	Temperature	120 ^{0}C
	Spray angle	75 degree
	Solvent composition	98% methanol (*v*/*v*)
	Solvent flow rate	3 µL/min
	Nebulizing nitrogen gas pressure	0.4 MPa
**DESI 2D stage parameters**	Pixel size (X x Y)	50 µm x 50 µm
	Scanning speed	100 µm/sec (2 pixels/s)<br>400 µm/sec (8 pixels/s)
**Data acquisition parameters**	Polarity	Positive
	m/*z* range	150 to 550.
	Mass resolution	20000
	Mass window (Da)	0.02

s: second.

Table S4: Parameters used for the optimization and data acquisition by iMScope QT.

**Parameters**	**8 pixels/s**	**20 pixels/s**	**32 pixels/s**
Polarity	Positive	Positive	Positive
m/*z* range	150 to 550	150 to 550	150 to 550
Mass resolution	32000	32000	32000
Pixel size (X x Y)	50 μm x 50 μm	50 μm x 50 μm	25 μm x 25 μm
Laser diameter	2	2	2
Laser power	65	65	60
Laser shots	100	100	20
Repetition number (Hz)	1000	10000	10000
Detector voltage (kV)	2.4	2.4	2.4
Setting value of DL temperature (^{o}C)	300	300	300

s: second.
